# Use of Oral Contraceptives as a Potential Risk Factor for Breast Cancer: A Systematic Review and Meta-Analysis of Case-Control Studies Up to 2010

**DOI:** 10.3390/ijerph18094638

**Published:** 2021-04-27

**Authors:** Wiesław Kanadys, Agnieszka Barańska, Maria Malm, Agata Błaszczuk, Małgorzata Polz-Dacewicz, Mariola Janiszewska, Marian Jędrych

**Affiliations:** 1 Specialistic Medical Center “Czechów” in Lublin, 20-848 Lublin, Poland; wieslaw.kanadys@wp.pl; 2 Department of Medical Informatics and Statistics with E-learning Lab, Medical University, 20-090 Lublin, Poland; agnieszkabaranska@umlub.pl (A.B.); mariola.janiszewska@umlub.pl (M.J.); marian.jedrych@umlub.pl (M.J.); 3 Department of Virology with SARS Laboratory, Medical University, 20-093 Lublin, Poland; agata.blaszczuk@umlub.pl (A.B.); malgorzata.polz.dacewicz@umlub.pl (M.P.-D.)

**Keywords:** breast cancer, cancer risk, prevention, oral contraceptives, meta-analysis

## Abstract

Despite numerous studies evaluating the risk of breast cancer among oral contraception users, the effect of oral contraceptive on developing breast cancer remains inconclusive. Therefore, we conducted a systematic review of literature with meta-analysis in order to quantitative estimate this association. The bibliographic database MEDLINE and EMBASE, and reference lists of identified articles were searched, with no language restrictions, from the start of publication to August 2010. We performed a reanalysis and overall estimate of 79 case-control studies conducted between 1960–2010, including a total of 72,030 incidents, histologically confirmed cases of breast cancer and 123,650 population/hospital controls. A decrease was observed in cancer risk in OC users before age 25 years (0.91, 0.83–1.00). However, the use of OCs before the first full-term pregnancy had a significant increased risk of breast cancer (OR, 1.14, 1.01–1.28, *p* = 0.04), as did OC use longer than 5 years (1.09, 1.01–1.18, *p* = 0.02). Pooled crude odds ratios of breast cancer in ever-users of oral contraceptives was 1.01 [95% confidence interval (CI), 0.95–1.07], compared with never-users. There was no significant increase in risk among premenopausal women (1.06, 0.92–1.22), postmenopausal women (0.99, 0.89–1.10), or nulliparous women (1.02, 0.82–1.26). Oral contraceptives do not appear to increase the risk of breast cancer among users. However, OC use before a first full-term pregnancy or using them longer than 5 years can modify the development of the breast cancer.

## 1. Introduction

Breast cancer (BrCa) is the most frequently diagnosed cancer and the leading cause of cancer death in women worldwide. It accounts for 23% (1.38 million) of the total new cancer cases and 14% (458,400) of the total deaths in 2008. About half the BrCa cases and 60% of the deaths occur in economically developing countries [1]. Of every thousand women aged 50, two will recently have given a diagnosis of BrCa, and about 15 will have had a diagnosis made before age 50, giving a prevalence of BrCa of nearly 2% [2].

In Western countries, the incidence has increased BrCa over the last 50 years, and these increases were dramatic in both the 1980s and the 1990s [3,4]. The sharp incidence increases observed after 1985 in the United States and most European countries were mainly due to the combined effects of changes in reproductive factors, prevalence of overweight and obesity, improvements screening mammography, and use of hormone replacement therapy (HRT) [5]. After peaking in 2000, the incidence of invasive BrCa steadily decreased, as did the mortality rate [6,7]. The potential explanation for the observed decrease in incidence may be the decline in the use of HRT after the publication of the results of the Women’s Health Initiative study in 2002 [7,8]; the early detection and management of invasive and precancerous breast lesions and the subsequent increase in management of these lesions with selective estrogen receptor modulators; and observed changes in lifestyle, such as increased exercise, decreased obesity, and dietary habits [9]. It may also be the result of the recently observed reduction in mammographic screening, which may lead to a decrease in BrCa detection among unscreened women [10].

The etiology BrCa is complex. Susceptibility is influenced by both environmental and genetic factors. In 5–10% of the cases, BrCa has a hereditary basis. BrCa susceptibility is generally inherited as an autosomal dominant with limited penetrance. In 15–30% of patients from high-risk families, BrCa is caused by a germline mutation in the BRCA1 or BRCA2 gene [11]. Many risk factors are associated with the development of BrCa, but the causal mechanism cannot be directly linked to a single one of them which include: age, familial history of BrCa, personal history of benign breast biopsy (atypical ductal and lobular hyperplasia—ADH and ALH), breast density, factors associated with lifestyle (cigarette smoking, alcohol use, dietary fat intake, and postmenopausal obesity), and reproductive factors (nulliparity, late age of first live birth) [2]. Experimental and epidemiological evidence suggests that exposure to endogenous hormones, notably estrogens and androgens, promotes breast carcinogenesis. Factors related to increased estrogen exposure throughout a woman’s lifetime, such as early menarche, late menopause, use of oral contraceptives, and hormone replacement therapy, have been associated with a ~2-fold increase in BrCa risk among women [12,13].

Oral contraceptives (OCs) among the most widely used methods are of effective and reversible family planning [14]. Many questions exist concerning a possible association between the use of OCs and the development of cancer. Benefits and risks of OCs use on cancer were reviewed by Working Groups of the International Agency for Research on Cancer (IARC/WHO) in 2007, which concluded that OC are carcinogenic to humans [15]. OCs use has been associated with an excess risk of benign liver tumors and modest increased risk of liver cancer and cervical cancer. Simultaneously OC protect again the risk of ovarian and endometrial cancer. No association is present between OC use and lung, other digestive tract neoplasms, cutaneous malignant melanoma, thyroid cancer, and any of the other neoplasms investigated [16].

Despite the large number of studies in this area, no consensus is present regarding the exact effect of OCs on the risk of BrCa [9,17]. In this context, we sought to evaluate whether women using oral contraceptives exhibit an increased risk of BrCa by performing a systematic review and meta-analysis of the relevant data from available case-control studies.

## 2. Materials and Methods

### 2.1. Search Strategy

We systematically searched the PubMed (Medline) and EMBASE bibliographic databases (from their commencements to August 2010), with no language restriction, to identify studies investigating the relation between OC uses and the risk of BrCa developing. For computer searches, we used the following MeSH terms or keywords: ’breast cancer’, ‘breast malignancy’ or ‘breast neoplasm’, combined with ‘oral contraceptives’, ‘contraceptive agents’ or ‘birth control pill’. In addition, we examined the references of identified articles, previous review articles and meta-analysis, and other relevant publications to identify further pertinent studies [18,19,20,21,22].

### 2.2. Study Selection

We selected studies in a two-stage process. Initially, a preliminary selection of articles was made after the electronic searching of titles. When the citation was relevant or when title/abstract was not sufficient for inclusion/exclusion, full-texts were retrieved and evaluated. Secondly, full manuscripts of selected citation and the additional items derived from a review of literature of selected papers and review articles were obtained. Final inclusion/exclusion decisions were made after independent and duplicate examination of the full manuscripts.

**Figure 1:**
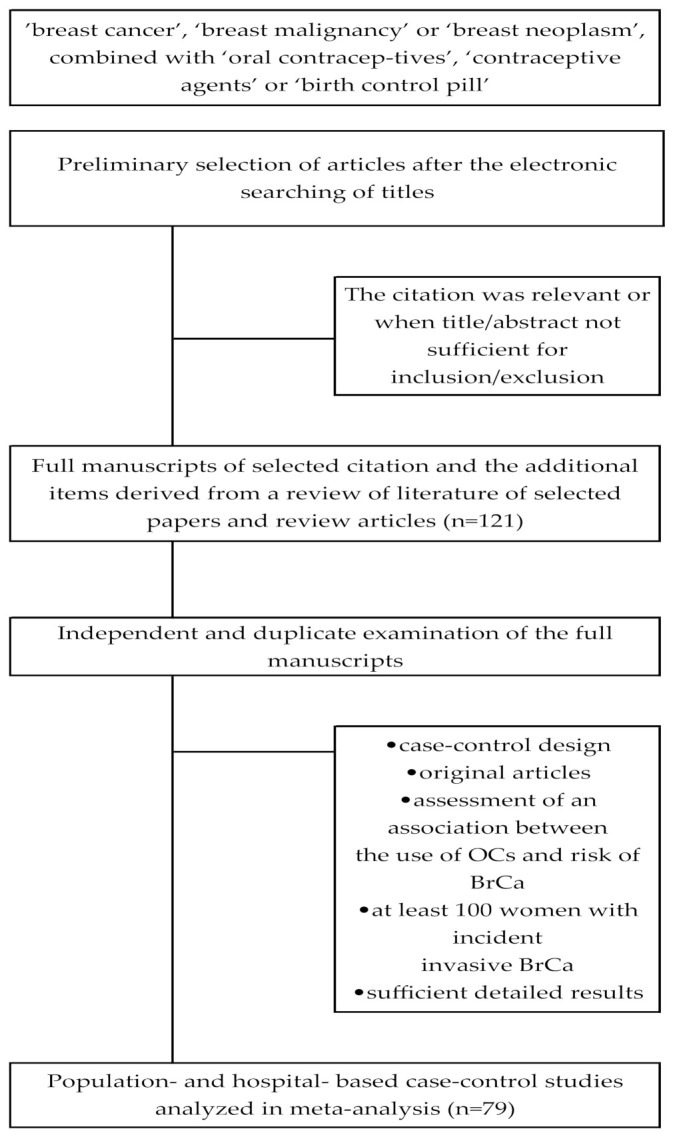
Flowchart of the selection procedure for studies included in meta-analysis regarding use of oral contraceptives as a potential risk factor for breast cancer.

Studies were included in the systematic review if they were a case-control design (population- and hospital-based) and published as original articles that permitted assessment of an association between the use of OCs and risk of BrCa. Studies eligible for meta-analysis fulfilled the following criteria: including at least 100 women with incident invasive BrCa, and presenting sufficient detailed results to calculate the odds ratio (OR) and 95% confidence interval (CI). In the case of multiple published studies based on the same population or subpopulation, we took into account only the most recent published report from the study with the longest period of recruitment, unless the earlier reports contained information not available in subsequent reports or represented a comprehensive form of this study. The selection procedure for studies included in meta-analysis is presented in Figure 1.

### 2.3. Data Extraction

The following data were extracted for each study: (a) clinical and methodological study characteristics such as last name of first author, publication year, country in which the study was performed, name of the study, years of data collection, number of cases and controls subjects, and source of cases; (b) information on use of OC (exposure)—ever/never use, duration of use at least above 5 years, use before first full-term pregnancy, and use in different age groups (<25 years, premenopausal, and postmenopausal women). To summarize the results associated with menopausal status, we adopted the following definitions: (a) premenopausal women or women younger than 50 years and (b) postmenopausal women or women older than 50 years. To assess the risk of developing breast cancer associated with the use of oral contraceptives with different composition (high- versus low formulation), we classified the available studies (based on the period of recruitment) to the following time ranges: 1966–1985 and 1986–2010 [23,24,25,26]. We additionally conducted a separate meta-analysis to determine whether the size of the risk of breast cancer is affected by the type of control group, based on the neighborhood/general population or on hospital.

If the study presented data on breast cancer only in subpopulations according to different ethnicity, age group, parity, etc., we conducted a cumulative estimate of these data for the entire study population. When the breast cancer event rate, OC use rate or control recruitment rate was expressed as a percentage, the number of patients was calculated by multiplying the rate by the total number of women in the relevant group. When data were expressed in a way that did not allow exact figures to be obtained, these data were not included in the meta-analysis.

### 2.4. Assessment of Study Quality

The Newcastle–Ottawa Scale (NOS) score was employed to evaluate the study quality of observational studies, with a maximum score of 9, of which 0–3, 4–6, and 7–9 scores were considered as low, fair, and high quality, respectively [27]. With this tool, each study in the meta-analysis was assessed in three separate categories: selection of cases and controls, comparability of cases and controls on the basis of the design or analysis, and ascertainment of exposure. The NOS quality stars ranged between 5–7, and the average score was 5.5 for case-control studies (Supplementary Materials File S1). Seven (7.86%) studies were regarded as high quality (NOS ≥ 7 points).

### 2.5. Statistical Methods

Meta-analysis of summary statistics from individual studies was performed utilizing the Medical Package program of STATISTICA 11.0 software (StatSoft Poland, Krakow, Poland). For each study, we constructed separate two-by-two (2 × 2) contingency tables to calculate the odds ratios (OR) and 95% confidence intervals (CIs), cross-classifying OC users and occurrence of breast cancer. The Mantel-Haenszel test was calculated to assess the association between OC use and cancer. An OR of one indicates that the odds of having breast cancer are the same in the case group and the control group; an OR greater than one indicates that the odds of having breast cancer in the case group are greater than in the control group; an OR of less than one indicates that the odds of having breast cancer in the control group are greater than in the case group. Because about one-third of the studies did not present adjusted ORs, only crude ORs were used in the primary meta-analysis. Meta-analyses combining the ORs across studies were conducted using the DerSimonian–Laird random effects model [28]. The random effects meta-analysis model was applied due to the diversity of research in terms of, for example, design and population. In the random effects model, it is assumed that there is no common effect size for independent studies. Instead, each study is assumed to have a different population effect size, which is a random variable and has a normal distribution. Therefore, there is a difference between the size of the effects of individual studies. Thus, the variance of the effect size in the random effects model is the sum of the variance within and between studies. As suggested by DerSimonian and Laird [28], the variance ‘between studies’ is estimated using the moments method. Weighting of the studies in the meta-analysis was calculated on the basis of the inverse of the sum of ‘within study’ and between studies variances. Heterogeneity was assessed graphically using forest plots and statistically using the Q test and I2 index. I2 values of 25%, 50%, and 75% were regarded as respectively representing low, moderate, and high heterogeneity between studies [29,30].

Due to the high heterogeneity of the studies, the following subgroups were also analyzed: case-control studies of the period of recruitment into the study before 1986/case-control studies of the period of recruitment into the study after 1986; premenopausal women or women younger than 50 years/postmenopausal women or women older than 50 years/women under 25 years old; nulliparous women; ever use of oral contraceptives before first pregnancy; and ever use of oral contraceptives for longer than 5 years.

For all the analyses, forest plots were generated to display results, whereby diamonds represent study-specific odds ratios and 95% CIs for individual studies are represented by horizontal lines.

To analyze the publication bias, the Begg and Mazumdar and Egger tests were performed. The meta-analysis methodology was based on the guidelines of DerSimonian and Laird [28] and Higgins et al. [29,30]. The method used is globally recognized as the primary method for the evaluation of case-control studies.

## 3. Results

Our systematic review included 121 population- and hospital-based case-control studies that analyzed the relationship between use of oral contraceptives and histologically confirmed breast cancer. Of these, 79 that met inclusion criteria qualified for the meta-analysis [31,32,33,34,35,36,37,38,39,40,41,42,43,44,45,46,47,48,49,50,51,52,53,54,55,56,57,58,59,60,61,62,63,64,65,66,67,68,69,70,71,72,73,74,75,76,77,78,79,80,81,82,83,84,85,86,87,88,89,90,91,92,93,94,95,96,97,98,99,100,101,102,103,104,105,106,107,108,109].

### 3.1. Description of the Studies

Table 1 shows papers in chronological order of recruitment period. Study periods included years between 1960–2010. All papers were published in English, except for one in Portuguese [95]. The total sample included 72,030 women with breast cancer and 123,650 women without breast cancer; respectively, of this number, 32,326 women (44.8%) and 53,365 women (43.2%) ever using combined oral contraceptives. North American studies were most common (n = 30), followed by 20 from Europe, 9 from Asia-Pacific, 7 from West Asia, 5 from Australia/New Zealand, 3 from Africa, 2 from South America, and 1 from Central America. One study was conducted in Sweden and Norway [68], and the WHO Collaborative Study of Neoplasia and Steroid Contraceptives [58] analyzed multinational data (participating countries: Australia, Chile, Republic of China, Colombia, the German Democratic Republic, Israel, Kenya, Mexico, Philippines and Thailand). In 39 studies, cases sources were regional cancer registries, while in the remaining 40 studies, hospital data was used as the case source. Forty studies were based on neighborhood/general population control group, 32 studies based on hospital control groups, and 6 studies were based on clinic control groups. One study used a combination of hospital and population controls [95].

### 3.2. Summary Analysis

Of the 79 case-control studies, 17 reported a significant positive relationship for OC use and risk of BrCa [40,46,49,50,60,66,68,80,81,82,84,91,92,101,104,105,109], and a further 26 reported a positive but non-significant association [31,38,45,54,55,57,58,61,67,69,70,72,73,75,77,78,79,83,86,87,88,90,99,100,108]. In contrast, nine studies reported a significant inverse association [33,36,41,64,76,85,93,98,102], and 27 reported a non-significant inverse association [32,34,35,37,39,42,43,44,45,47,48,51,52,53,56,59,62,63,65,71,74,89,94,96,97,103,106,107]. Tessaro et al. [95] found a small increase in BrCa risk in a study using the control group of population (OR, 1.06; 95% CI, 0.72–1.57), and reduced risk with a control group of hospital patients (OR, 0.86; 95% CI, 0.57–1.29).

**Figure 2:**
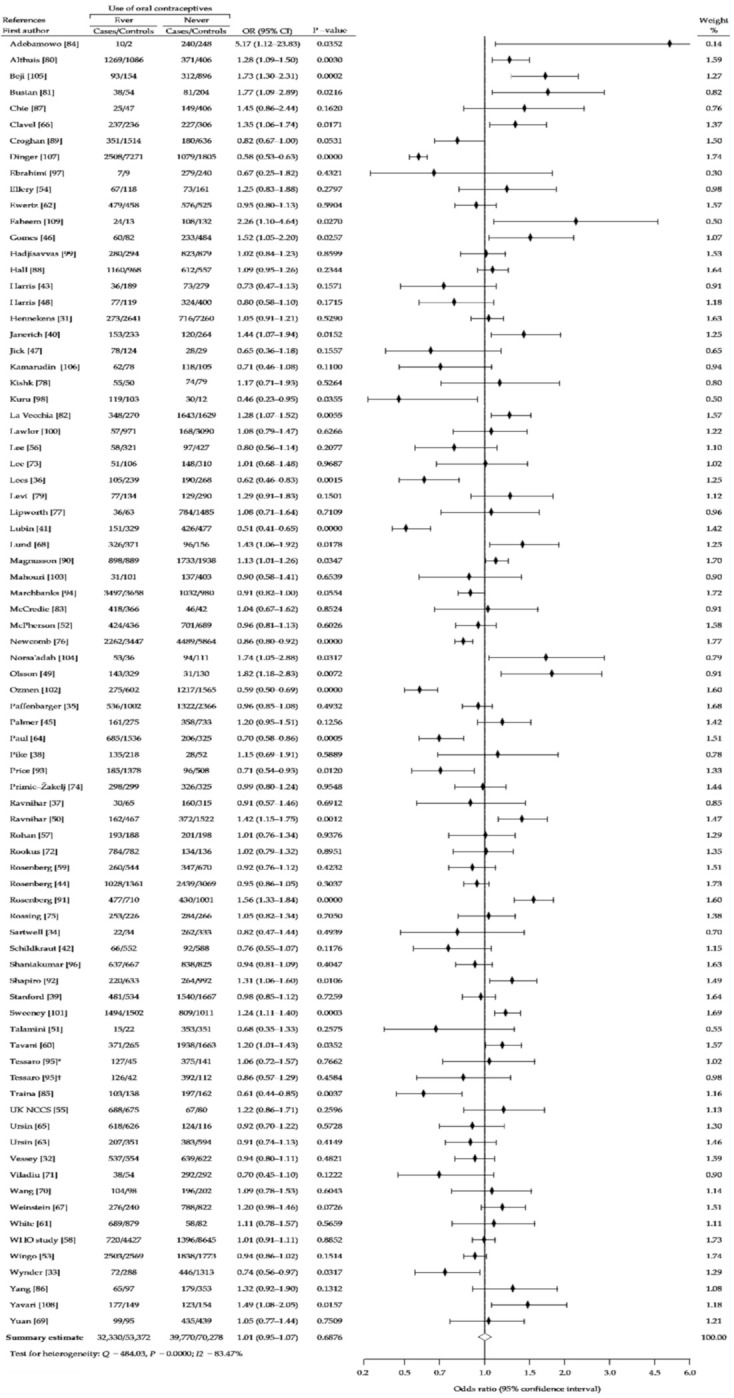
Forest plot and summary odds ratios on the association between risk of breast cancer and ever use of oral contraceptives: case-control studies conducted between 1960–2008, in alphabetical order. UK NCCS—UK National Case-control Study, WHO—World Health Organization, * neighborhood controls, ^†^ hospital controls.

**Figure 3:**
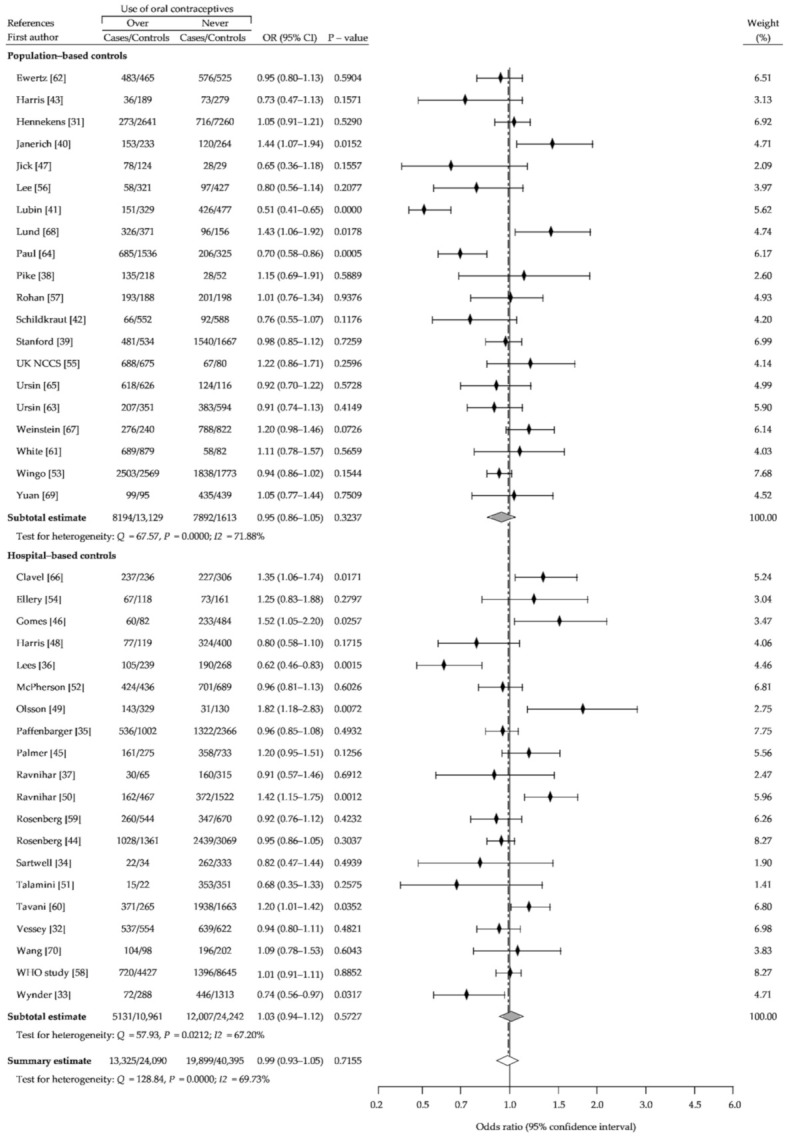
Forest plot and summary odds ratios on the association between risk of breast cancer and ever use of oral contraceptives: case-control studies of the period of recruitment into the study before 1986, in alphabetical order. UK NCCS—UK National Case-control Study, WHO—World Health Organization.

**Figure 4:**
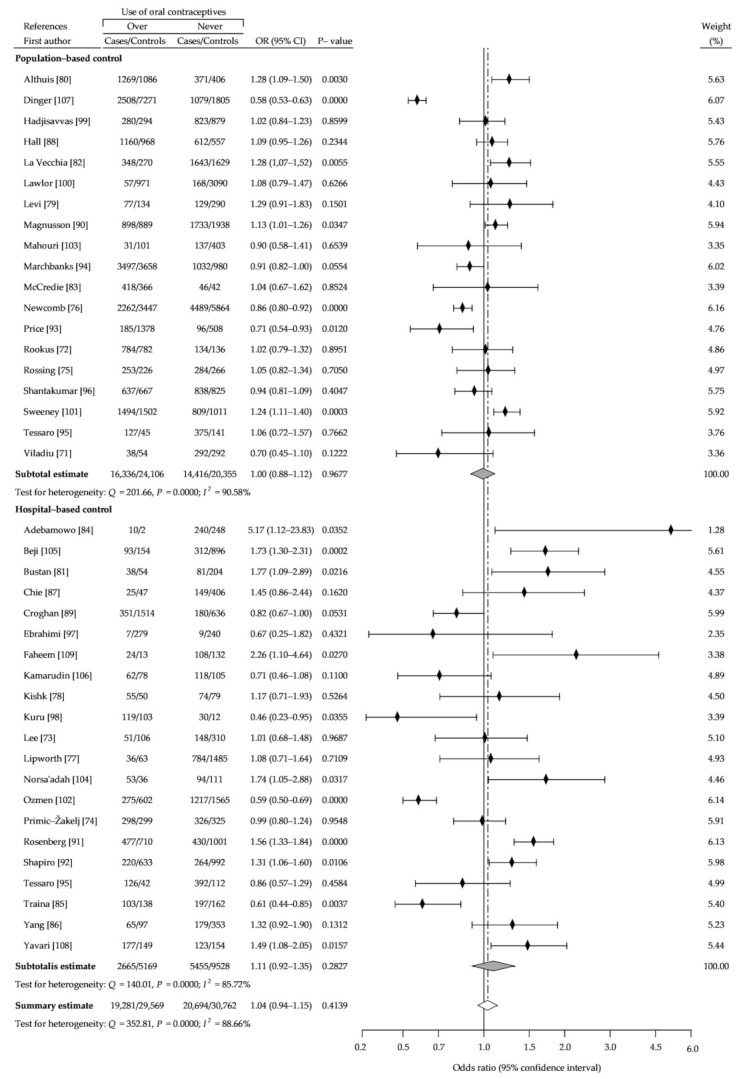
Forest plot and summary odds ratios on the association between risk of breast cancer and ever use of oral contraceptives: case-control studies of the period of recruitment into the study after 1986, in alphabetical order.

The present meta-analysis based on reanalysis and summary analysis of available data showed no difference in the association between OC ever-use and breast cancer risk, compared with never-use (crude OR (cOR), 1.01; 95% CI, 0.95–1.07; *p* = 0.69) (Figure 2). The Begg-Mazumdar and Egger tests showed no publication bias (*p* > 0.05). In addition, we performed separate analysis for assessment of relationship between risk of breast cancer and use of older oral contraceptives formulations containing higher doses of estrogen and oral contraceptives using low-estrogen formulations, based on the periods of recruitment to the study. Figure 3 (1966–1985) and Figure 4 (1986–2010) showed that in a studied subgroup, the overall estimates of BrCa risk slightly oscillated around the unit: cOR = 0.99 (95% CI, 0.93–1.05; *p* = 0.71) and cOR = 1.04 (95% CI, 0.94–1.53; *p* = 0.41), respectively. Similar results were obtained for the matched control groups in the analyzed subgroups (Figure 2 and Figure 3).

**Figure 5:**
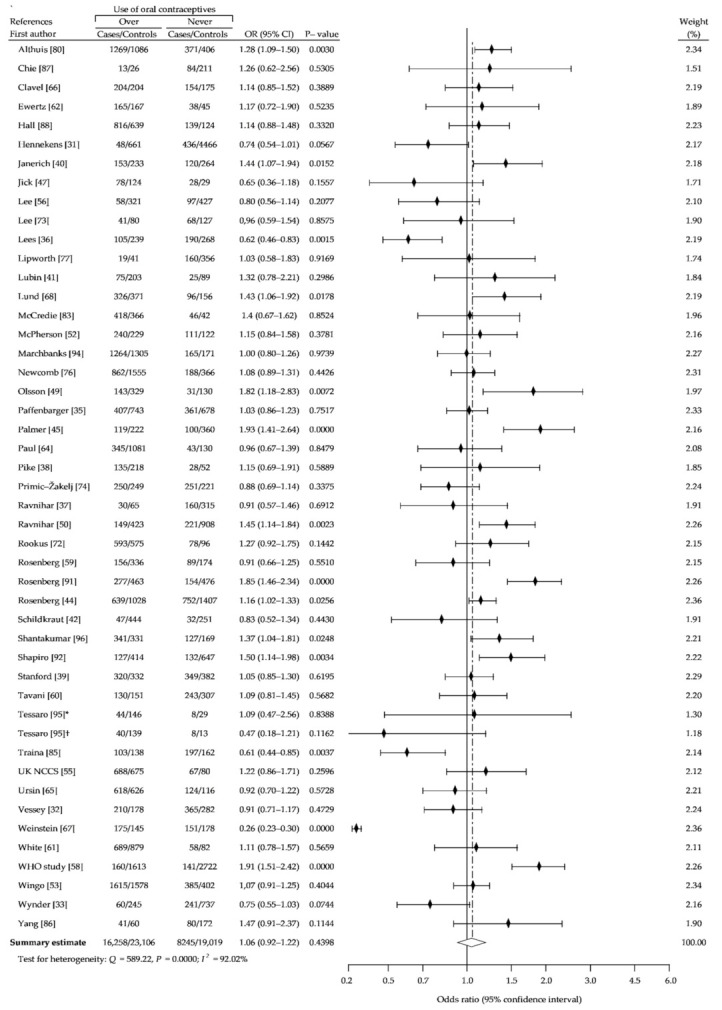
Forest plot and summary odds ratios on the association between risk of breast cancer and ever use of oral contraceptives in premenopausal women or women younger than 50 years: case-control studies in alphabetical order. UK NCCS—UK National Case-control Study, WHO—World Health Organization, * neighborhood controls, ^†^ hospital controls.

**Figure 6:**
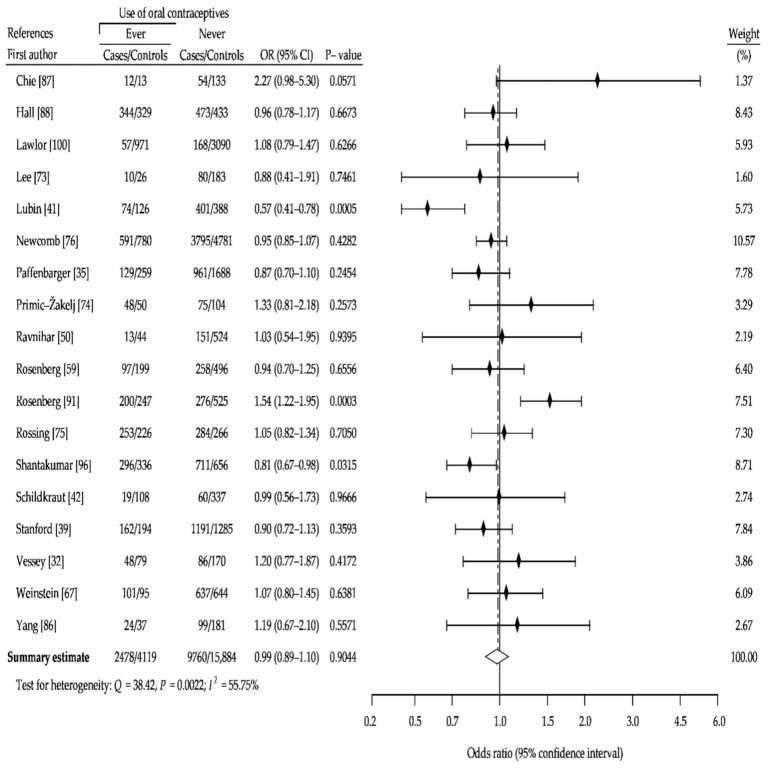
Forest plot and summary odds ratios on the association between risk of breast cancer and ever use of oral contraceptives in postmenopausal women or women older than 50 years: case-control studies in alphabetical order.

**Figure 7:**
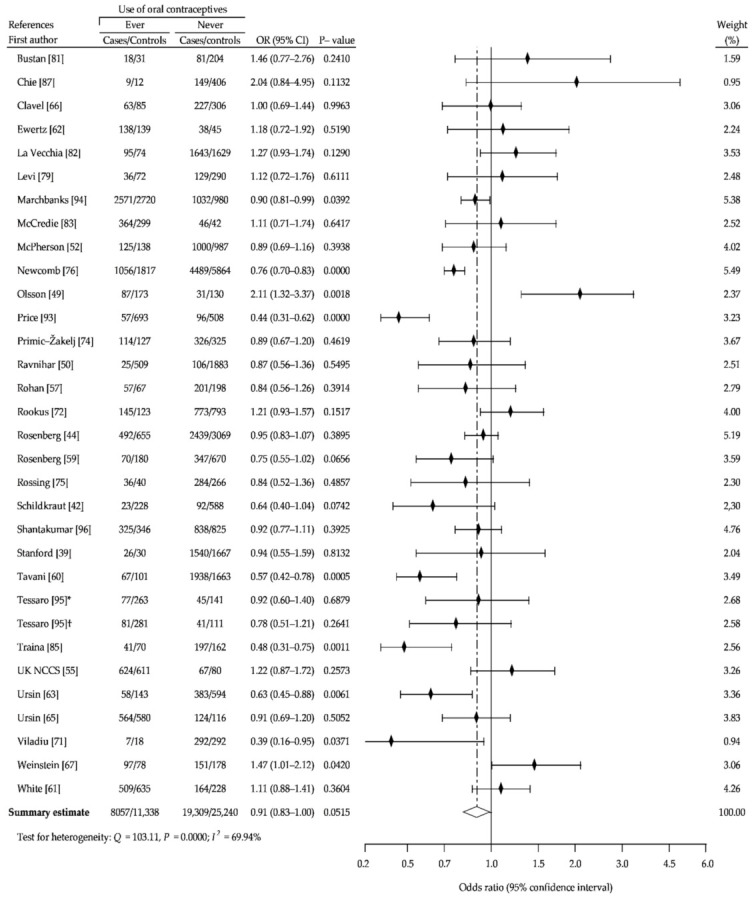
Forest plot and summary odds ratios on the association between risk of breast cancer and ever use of oral contraceptives in women under 25 years old: case-control studies in alphabetical order. UK NCCS—UK National Case-control Study, * neighborhood controls, ^†^ hospital controls.

Our analysis was restricted to premenopausal or women younger than 50 years and showed a marginal, non-significant increase in risk of breast cancer with ever use of OCs (cOR, 1.06; 95% CI, 0.92–1.22; *p* = 0.44), compared with controls that had never used OCs (Figure 5). Analysis of risk among postmenopausal or women over age 50 ever using OCs also did not show difference (cOR, 0.99; (95% CI, 0.89–1.10; *p* = 0.90) (Figure 6). Moreover, we indicated a small decrease in risk of developing breast cancer in OC users who began before age 25 years (cOR, 0.91; 95% CI, 0.83–1.00; *p* = 0,05) (Figure 7).

**Figure 8:**
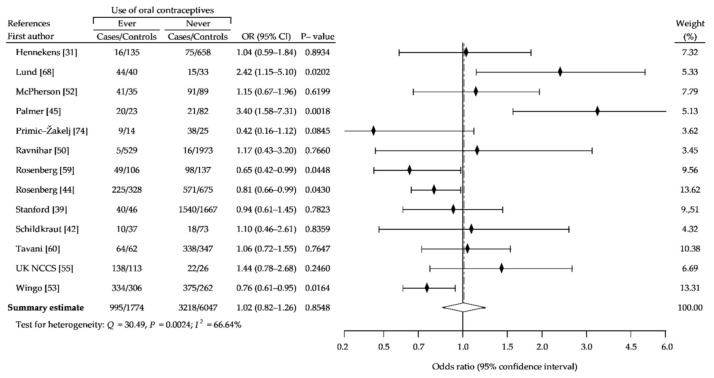
Forest plot and summary odds ratios on the association between risk of breast cancer and ever use of oral contraceptives use in nulliparous women: case-control studies in alphabetical order. UK NCCS—UK National Case-control Study.

**Figure 9:**
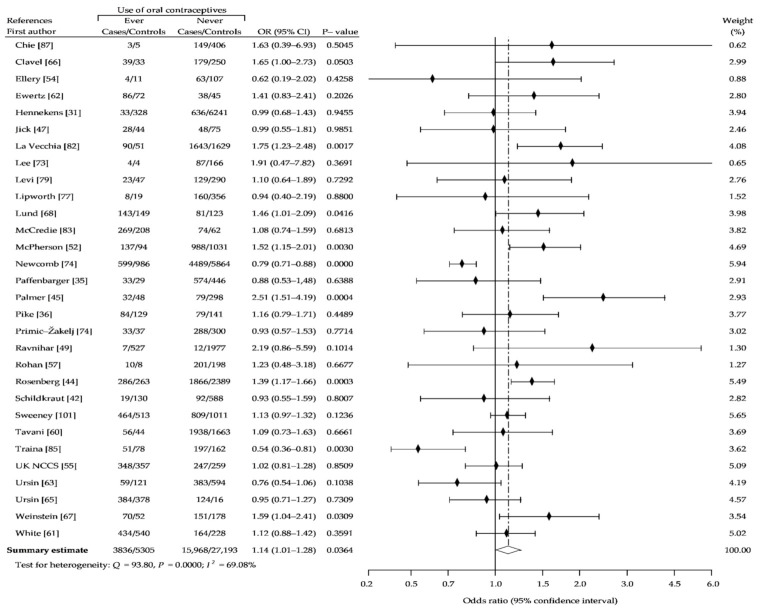
Forest plot and summary odds ratios on the association between risk of breast cancer and ever use of oral contraceptives use before first pregnancy: case-control studies in alphabetical order. UK NCCS—UK National Case-control Study.

**Figure 10:**
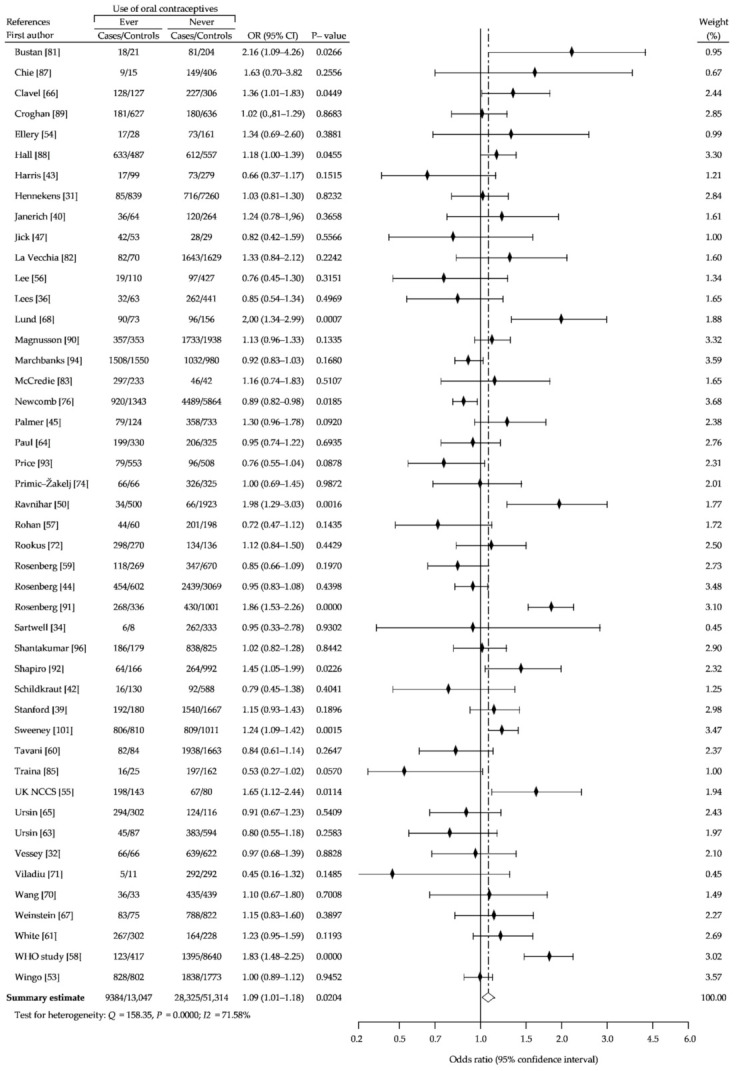
Forest plot and summary odds ratios on the association between risk of breast cancer and ever use of oral contraceptives longer than 5 years: case-control studies in alphabetical order. UK NCCS—UK National Case-control Study, WHO—World Health Organization.

As shown in Figure 8, the use of OC by nulliparous women did not affect breast cancer risk (cOR, 1.02; 95% CI, 0.82–1.26: *p* = 0.85), although the use of pills before a first full-term pregnancy has a significant effect on increased risk of breast cancer (cOR, 1.14; 95% CI, 1.01–1.28; *p* = 0.04), compared with never-use (Figure 9). Furthermore, OC use longer than 5 years leads to a slight, but significant increase of breast cancer risk (cOR, 1.09; 95% CI, 1.01–1.18; *p* = 0.02) (Figure 10).

## 4. Discussion

Despite numerous epidemiological studies that have attempted to assess the risk of breast cancer among oral contraception users, the effect of oral contraceptive use on the developing breast cancer remains controversial. Several meta-analyses and a very large pooled analysis have addressed this issue. Romieu et al. [110], in meta-analysis based on 27 case-control studies published in 1966–1989, showed a marginally, non-significant increase in the risk of breast cancer among women ever using oral contraceptives [Relative Risk (RR), 1.06; 95% CI, 0.98–1.14)). Delgado-Rodriguez et al. [20], in their meta-analysis including 40 case-control and 7 cohort studies published between 1966–1990, found that OC use was associated with slightly, but significantly, increased BrCa risk (RR, 1.06; 95% CI, 1.02–1.10). In the same year, Thomas [111] presented a meta-analysis of data from 17 case-control studies published from 1974–1990. This author found no differences in risk of BrCa among ever- and never-users of oral contraceptives (RR, 1.0; 95% CI, 1.0–1.1). Rushton and Jones [19], analyzing data from 21 case-control studies from the years 1980–1989, showed that the overall relative risk was 1.12 (95% CI, 1.05–1.20). Kahlenborn et al. [22] performed a meta-analysis of 34 case control studies of the relationships between OC administration and premenopausal breast cancer published in or after 1980 and found oral contraceptive use was associated with an increased BrCa risk (OR, 1.19; 95% CI, 1.09–1.29). A pooled analysis made by Collaborative Group on Hormonal Factors in Breast Cancer [21] of data from 54 epidemiological studies, reported in 1996, showed a small increase in the risk of BrCa with ever use (RR = 1.07 for all studies; RR = 1.07 for cohort studies; RR = 1.02 for case-control studies with population controls; RR = 1.17 for case-control studies with hospital controls). Our large standardized meta-analysis conducted on the largest data set from 79 case-control studies published from 1960–2010, showed that ever use of OC is not associated with an increased risk of breast cancer, compared with never using (OR = 1.1).

Evaluation of carcinogenic risks associated with changes in the formulations of OC over the past decades, especially of lowering estrogen content, also remains unclear. In our meta-analysis, based on accepted criteria, we found no increased risk of breast cancer associated with use of low-potency/low estrogen-dose oral contraceptives, a finding which was comparable with earlier high-potency/high-dose preparations. Our results confirmed earlier reports of Collaborative Group on Hormonal Factors in Breast Cancer [112] that among women for whom information was available concerning the OC formulations used, there was no significant variation in the breast cancer risk associated with use specific types and doses of estrogen or progestogen. In a meta-analysis based on different periods of inclusion in the study, Romieu et al. [110] also showed no significant effect of different OC formulations on risk of breast cancer: Cases accrued between 1975–1989—RR = 1.08, and cases accrued between 1980–1989—RR = 1.06. Rushton and Jones [19], based on the criterion of study ending, adopted a limit of 1982 or earlier versus 1983 or later, demonstrating that low-dose OCs are associated with a greater risk of breast cancer than high-dose regimens, RR = 0.98 and RR = 1.16, respectively. These differences were more pronounced, depending on the age of women using OC: RR = 0.90 and RR = 1.25, respectively for age <45 years, and RR = 1.06 and RR = 1.00, respectively for age ≥45 years. Similarly, Kahlenborn et al. [22] also showed an increased risk of premenopausal breast cancer under the influence of low-dose OC (OR = 1.19). 

In our meta-analysis, we performed only separate analysis for assessment of relationship between risk of breast cancer and use of older oral contraceptives formulations containing higher doses of estrogen and oral contraceptives using low-estrogen formulations, based on the periods of recruitment to the study, creating two subgroups—the periods of recruitment 1966–1985 and 1986–2010. However, we have not identified different oral contraceptives, because only 14 of the 79 studies included in the meta-analysis reported the type of oral contraceptive used. The analysis of more than half of these studies showed that no statistically significant differences were found in the incidence of breast cancer between cases and controls, depending on the use of different oral contraceptives [32,43,44,47,50,62,65,94]. In contrast, White et al., demonstrated that the use of high-progestin pills is associated with an increased risk of breast cancer on use for 1–5 years (OR = 1.64; 95% CI = 1.14–2.35) or 6 years and above (OR = 1.61; 95% CI = 1.0–2.60) [61]. A study by Rookus et al., found an increased risk for the use of low-dose estrogen pills over 8 years (RR = 3.0; 95% CI = 1.5–6.1) and norethisterone (acetate) pills over 4 years of use (RR = 2.0; 95% CI = 1.0–4.1) or ever used desogestrel-containing pills (RR = 1.7; 95% CI = 1.1–2.5) [72]. Furthermore, the UK National Case-Control Study has shown an increase in the risk of breast cancer with high estrogen pills over time (*p* < 0.001) [55]. On the other hand, McPherson et al., showed an increased risk of breast cancer when using ethinylestradiol over 48 months before the first pregnancy (RR = 2.62; 95% CI = 1.15–5.95 [52]. In turn, in the work of Althuis et al., the use of several types of oral contraception was associated with an increased risk of breast cancer [80]. The highest risk was associated with the use of ethynodiol diacetate (RR = 12.0; 95% CI = 2.4–59.2), high doses of progestin (RR = 8.11; 95% CI = 2.1–31.6), and a combination of high doses of progestin and low doses of estrogen (RR = 8.07; 95% CI = 2.1–31.4). Finally, the Shapiro et al., study indicated an increased risk of current use of injectable progestogen contraceptives (RR = 1.6; 95% CI = 1.1–2.3) [92].

Results of our meta-analysis did not always disagree with those from previous reports suggesting that certain groups of women have an increased risk of breast cancer related to OC use. We failed to show appreciable differences in the risk of breast cancer between women who had taken OC and those who had not when we sub-grouped women according to menopausal status. Analysis of cancer risk in premenopausal women showed a slight, statistically non-significant increase (OR, 1.06; 95% CI, 0.92–1.22) and a marginal decrease in postmenopausal women (OR, 0.99; 95% CI, 0.89–1.10). In contrast, Romieu et al. [110] showed that each use of OC increases, but non-significantly, breast cancer risk among women ≤45 years of age (RR, 1.17; 95% CI, 0.95–1.45). A similar result was obtained by Delgado et al. [20]—RR = 1.10. Rushton and Jones’s meta-analysis [19] demonstrated a statistically significant increase in risk in women <45 years (RR, 1.16; 95% CI, 1.07–1.25) and a marginal increase in women ≥45 years (RR, 1.03; 95% CI, 0.4–1.13). These results were confirmed by Kahlenborn et al. [22] (OR, 1.19; 95% CI, 1.09–1.29), as well as by Thomas [111] (RR, 1.16; 95% CI, 1.05–1.28). 

The controversy also concerns breast cancer risk in young women taking the pill. In our investigation, among women who started using OC at an early age (before 25 years), we saw a slight decrease in risk of breast cancer (OR 0.91, 95% CI, 0.83–1.00). By contrast, the meta-analyses that analyzed risk factor for age-related use reported that an early start in taking birth control pills was associated with the risk of cancer. In the Oxford study [21], the authors showed that in users <20 years, the RR was 1.22 and for 20–24 years it was 1.04. Delgado-Rodriquez et al. [20] also demonstrated increased risk under 25 years (RR, 1.25; 95% CI, 1.10–1.44).

The results of our analysis (OR, 1.02) do not confirm previous papers that reported an increased RR in the range of 1.21–1.30 for oral contraception use by nulliparous women [19,21,22]. One of the important findings of our meta-analysis, however, was to demonstrate an increased risk of breast cancer among ever users of oral contraceptives before first full-term pregnancy compared with never users (OR, 1.14; 95% CI. 1.01–1.28). The Oxford pooled analysis found that current OC users had a higher cancer risk (RR, 1.33) [21]. This also confirmed the results of earlier meta-analyses, which showed an increased risk of breast cancer in these women: Romieu et al. [110]—RR = 1.72 (95% CI, 1.36–2.19), Delgado-Rodriguez et al. [20]—RR = 1.17 (95% CI, 1.06–1.30), Thomas [111]—RR = 1.4 (95% CI, 1.2–1.7), and Kahlenborn et al. [22]—OR = 1.44 (95% CI, 1.28–1.62).

We revealed in our meta-analysis that OC use longer than 5 years leads to a modest, but significant increased risk of breast cancer (OR, 1.09; 95% CI, 1.01–1.18). Romieu et al. [110] also showed that OC use for 10 years or longer increased cancer risk (RR, 1.14; 95% CI, 0.90–1.42). Similarly, Thomas [111] demonstrated that summary relative risk of breast cancer in women under 45 years who used OC for a long period (≥10 years) was 1.42 (95% CI, 1.25–1.63). In turn, Rushton and Jones [19] in their meta-analysis estimated the RR = 1.27 (95% CI, 1.12–1.44) for use durations of more than 8 years. The Oxford analysis [21] found that relative risk of breast cancer by 5–9 years duration of OC use 5–9 years was 1.09, by duration of 10–14 years was 1.6, and by ≥15 was 1.08.

Very important discoveries regarding the etiology of breast cancer concern the mutations in the BRCA1/2 genes. However, none of the works in this meta-analysis have tested cases and controls in this regard. Indeed, only Ursin et al., and Marchbanks et al., mentioned in their discussion sections the possible influence of BRCA1 and BRCA2 mutations on the occurrence of breast cancer with the simultaneous use of OC [65,94]. It should also be noted that Beji and Reis analyzed the occurrence of breast cancer in the context of family history, writing that BRCA1/2 mutations are found in most families with a positive history of breast, ovarian, and endometrial cancer [105]. However, they add that the people taking part in the study were not tested for the presence of these mutations. In turn, Mahouri et al., mentioned the need to study the impact of mutations in the BRCA1 and BRCA2 genes on the incidence of breast cancer in Iranian women [103]. 

In the period analyzed by us (up to 2010), however, there were several papers dealing with the topic of mutations in the BRCA1 and BRCA2 genes. Brohet et al., showed that BRCA1/2 mutation carriers who had ever used oral contraception had a significantly increased risk of breast cancer (HR = 1.47; 95% CI = 1.16–1.87). Longer use of OC, especially before the first full-term pregnancy, was also associated with an increased risk of breast cancer for both BRCA1 and BRCA2 mutation carriers (HR = 1.49; 95% CI = 1.05–2.11 for BRCA1 subjects and HR = 2.58; 95% CI = 1.21–5.49 for BRCA2 subjects) [113]. Still, in a study by Lee et al., no association was found between the use of oral contraceptives and the risk of breast cancer in carriers of BRCA1 and BRCA2 mutations [114]. Moreover, a study by Milne et al. showed that the use of oral contraceptives for at least 12 months significantly reduced the risk of breast cancer for BRCA1 mutation carriers (OR = 0.22; 95% CI = 0.10–0.49), but not for BRCA2 mutation carriers (OR = 0.93; 95% CI = 0.69–1.24) [115].

### Study Limitations

In our meta-analysis, significant heterogeneity between studies is observed, although the specific cause of its occurrence across individual studies could not be easily explained.

We were aware of a number of limitations that may affect the interpretation of our results. First, as with all meta-analyses, the validity of the results is limited by the conduct and reporting of the studies from which the data were extracted and pooled. In order to reduce the possibility of publication bias, we limited the search to studies published in indexed journals, and we did not search for non-published studies. Nevertheless, lack of these reports as well as the possibility of not reaching out to all publications in this topic may affect the value of the results [116]. Secondly, the studies contributing to summary estimates are vulnerable to various types of bias. Retrospective self-reporting of users of oral contraceptives was present in the studies we found; this may be associated with overestimation or underestimation of data. Yet another element of bias that must be considered is the possibility to make a mistake in recruitment to the control groups, especially based on hospital populations. Selection bias can also occur when cases are either more or less likely than controls to be selected for study depending on their use of OC. Another possible source of bias present in meta-analysis is the lack of a uniform definition of ’ever’ use of oral contraceptives. This term contains a different period of exposure to OC, defined by the various times that pills were started and stopped. This may lead to misclassifications, which may weaken the true association between OC use and breast cancer (as it may occur unequally among cases and controls).

The limitations should also take into account other coexisting factors, such as the use of different oral contraceptives, as well as the presence of unexplored genetic factors, such as BRCA1 and BRCA2 mutations. We are, hence, currently preparing a meta-analysis in which we will examine the effect of BRCA1 and BRCA2 mutations on the occurrence of breast cancer.

A further limitation lies in the fact that most of the studies in this meta-analysis only look at the effect of contraception on breast cancer in general. Moreover, only some studies assessed the risk of breast cancer after using different oral contraceptives. These articles were too small a research area for the analyses we undertook. Furthermore, in all of the analyzed studies, participants were not tested for the presence of mutations in the BRCA1 and BRCA2 genes.

Finally, as with other meta-analyses, given that many studies are not included in the evaluation of additional variables or analyzed differing confounders, we were not able to adjust our overall analysis for these risk factors. Thus, we only used crude (not adjusted) study estimates in our meta-analysis. If there were strong effects from confounding factors, the estimates included in the meta-analysis might be biased. A prospective study needs to be conducted to confirm our findings.

## 5. Conclusions

In conclusion, the findings of our meta-analysis and systematic review suggest that oral contraceptives do not appear to increase the risk of breast cancer among women who are taking these preparations. However, OC use before a first full-term pregnancy or using them longer than 5 years can modify the development of the breast cancer.

## Figures and Tables

**Table 1:** Characteristics of the case-control studies included in the meta-analysis on the association between oral contraceptive use and breast cancer risk.

Study Name	Recruitment Period	Number of Case Subjects	Percent of OCs Users	Age Range	Source of Cases	Number of Controls Subjects	Percent of OCs Users	Source of Controls	NOS Sc.
Setting [Reference]				(Years)					
USA—Nationwide [31]	1960—1976	989	27.6	30–55	Population	9901	26.7	Population	5
UK—London, Oxford [32]	December 1968–September 1980	1176	45.7	16–50	Hospital	1176	47.1	Hospital	5
USA—New York City [33]	January 1969–December 1975	518	13.9	30<	Hospital	1601	18.0	Hospital	5
USA—Baltimore [34]	1969–1972	284	7.7	20–74	Hospital	367	9.3	Hospital	5
USA—San Francisco Bay Area [35]	January 1970–December 1972	1868	28.7	15–49+	Hospital	3391	29.6	Hospital	5
Canada—northern Alberta [36]	January 1971–December 1974	295	35.6	30–49	Hospital	507	47.1	Clinic	5
former Yugoslavia—Slovenia [37]	May 1972–November 1974	190	15.8	20–49	Hospital	380	17.1	Clinic	5
USA—Los Angeles County [38]	July 1972–December 1978	163	82.8	<33	Population	270	80.7	Population	5
Breast Cancer Detection Demonstration Project USA–Nationwide [39]	July 1973–November 1980	2022	23.8	<40–60+	Population	2183	24.5	Population	5
USA—New York State [40]	January 1974–August 1976	253	52.6	≤45	Population	497	46.9	Population	5
Canada—northern Alberta [41]	1976–1977	577	26.2	30–80	Population	826	42.3	Population	5
USA—North Carolina [42]	April 1977–December 1978	158	41.8	25–59	Hospital	1140	48.4	Population	5
USA—King County [43]	July 1977–August 1978	112	34.8	35–54	Population	466	39.7	Population	5
Case-Control Surveillance Study USA—Philadelphia. Baltimore, New York City [44,45]	1977–1992	3540	31.1	25–59	Hospital	4488	31.6	Hospital	7
		519	31.0	25–59	Hospital	1008	27.3	Hospital	5
Brazil—Belo Horizonte [46]	January 1978–December 1987	293	20.5	25–75	Hospital	566	14.5	Hospital	5
USA—Seattle [47]	July 1978–December 1983	127	61.4	<43	Population	174	71.3	Population	5
USA—New York City [48]	June 1979–February 1981	401	19.2	<30–70+	Hospital	519	22.9	Hospital	5
Sweden—southern region [49]	1979–1985	174	82.2	<45	Population	459	71.7	Population	5
former Yugoslavia—Slovenia [50]	January 1980–September 1983	534	30.3	24–54	Hospital	1989	23.9	Hospital	5
Italy—Pordenone [51]	January 1980–March 1983	368	4.1	27–79	Hospital	373	5.9	Hospital	5
UK—London. Oxford [52]	September 1980–1984	1125	37.7	16–64	Hospital	1125	38.8	Hospital	5
Cancer and Steroid Hormone Study USA—Atlanta. Connecticut, Detroit. Iowa, New Mexico. San Francisco. Seattle, Utach [53]	December 1980–December 1982	4341	57.7	20–54	Population	4343	59.2	Population	5
Australia—New South Wales [54]	1980–1982	141	48.2	25–64	Hospital	279	42.3	Hospital	5
UK National Case-Control Study UK—England and Scotland [55]	January 1982–December 1985	755	91.1	<35	Population	755	89.3	Clinic	6
Costa Rica [56]	January 1982–March1984	155	37.4	20–49	Population	748	42.9	Population	5
Australia—Adelaide [57]	April 1982–July 1984	395	48.9	20–69	Population	386	48.7	Population	5
WHO Collaborative Study of Neoplasia and Steroid Contraceptives * [58]	November 1982–February 1986	2116	34.0	20–59	Hospital	13072	33.9	Hospital	7
Canada—Toronto [59]	1982–1986	607	42.8	<69	Hospital	1214	44.8	Population	5
Italy—greater Milan area [60]	January 1983–December 1991	2309	16.1	22–59	Hospital	1928	13.7	Hospital	5
USA—Seattle metropolitan area [61]	January 1983–April 1990	747	92.2	21–45	Population	961	91.5	Population	6
Denmark [62]	March 1983–August 1984	1059	45.6	<59	Population	990	47.0	Population	5
USA—San Francisco-Oakland, Los Angeles, Oahu [63]	April 1983–June 1987	590	35.1	20–55	Population	945	37.1	Population	6
Auckland Breast Cancer Study New Zealand [64]	July 1983–June 1987	891	76.9	25–54	Population	1861	82.5	Population	6
USA—Los Angeles County [65]	July 1983–January 1989	744	83.1	<40	Population	744	84.1	Population	6
France–Strasburg, Lyons, Tours, Marseilles [66]	1983–1987	464	51.1	20–55	Hospital	542	43.5	Hospital	5
USA—Nassau and Suffolk County [67]	January 1984–December 1986	1420	25.9	20–79	Population	1420	22.6	Population	5
Sweden and Norway [68]	May 1984–May 1985	422	77.3	20–49	Population	527	70.4	Population	5
China—Shanghai [69]	June 1984–May 1985	534	18.5	20–69	Population	534	17.8	Population	5
China—Tiajin [70]	January 1985–November 1986	300	34.7	20–55	Hospital	300	32.7	Population	5
Spain—Girona [71]	July 1986–June 1993	330	11.5	<75	Population	346	18.5	Population	6
Netherlands Oral Contraceptives and Breast Cancer Study The Netherlands [72]	October 1986–June 1989	918	85.4	20–54	Population	918	85.2	Population	6
Singapore [73]	1986–1988	200	25.5	<40–70+	Hospital	420	25.2	Hospital	5
Slovenia [74]	January 1988–December 1990	624	47.8	25–54	Population	624	47.9	Population	6
USA—King County [75]	January 1988–June 1990	537	47.1	50–64	Population	492	45.9	Population	6
USA—Wisconsin, Maine, Massachusetts (excluding Boston), New Hampshire [76]	April 1988–December 1991	6751	35.5	<75	Population	9311	37.0	Population	5
Greece—Athens, Piareus [77]	January 1989–December 1991	820	4.4	56.4 ^†^	Hospital	1548	4.1	Hospital	6
Egypt [78]	n.a.	129	57.4	44.5 ^†^	Hospital	129	61.2	Hospital	5
Switzerland—Canton of Vaud [79]	January 1990–August 1995	206	37.4	27–75	Population	424	31.6	Hospital	6
USA—Atlanta, Seattle, New Jersey [80]	May 1990–December 1992	1640	77.4	20–44	Population	1492	72.8	Population	5
Indonesia [81]	1990–1991	119	31.9	25–55	Hospital	258	20.9	Hospital	5
Italy—Milan, Genoa, Naples, the provinces of Pordenone, Gorizia. Forli, Latina (near Rome) [82]	June 1991–February 1994	1991	17.5	23–64	Population	1899	14.2	Population	6
Australian Breast Cancer Family Study Australia—Melbourne, Sydney [83]	January 1992–July 1995	467	90.2	<40	Population	408	89.7	Population	5
Nigeria [84]	April 1992–December 1995	250	4.0	43 ^†^	Hospital	250	0.8	Hospital	5
Italy—Palermo, Turin [85]	1992–1994	300	34.3	<46	Hospital	300	46.0	Hospital	5
Taiwan—Taipei [86]	January 1993–December 1994	224	29.0	20–80	Hospital	450	21.6	Clinic	5
Taiwan—Taipei [87]	February 1993–June 1994	174	14.4	47.7 ^†^	Hospital	453	10.8	Hospital	5
Carolina Breast Cancer Study USA—North Carolina [88]	May 1993–December 2000	1778	65.6	20–74	Population	1535	63.7	Population	5
USA–Rochester [89]	August 1993–November 2003	531	66.1	58 ^†^	Hospital	2150	70.4	Clinic	6
Sweden [90]	October 1993–March 1995	3008	35.5	50–74	Population	3248	33.0	Population	5
Case-Control Surveillance Study USA—Philadelphia, Baltimore, New York [91]	1993–2007	907	52.6	25–69	Hospital	1711	41.5	Hospital	7
South Africa—Cape Town [92]	January 1994–October 1997	484	45.5	20–54	Hospital	1625	39.0	Hospital	7
Australia—New South Wales [93]	April 1994–April 1997	298	62.1	40–87	Population	1926	71.5	Population	5
Women’s Contraceptive and Reproductive Experiences Study USA—Atlanta, Detroit, Philadelphia, Los Angeles, Seattle [94]	July 1994–April 1998	4575	76.1	35–64	Population	4682	78.1	Population	5
Brazil–Pelotas [95]	March 1995–July 1998	172	73.8	20–60	Hospital	516	72.7	Hospital	6
		168	75.0			504	77.8	Population	
Long Island Breast Cancer Study Project USA—Long Island [96]	August 1996–July 1997	1475	43.2	20–98	Population	1492	44.7	Population	6
Iran—Tehran [97]	April 1997–April 1998	286	2.4	24–81	Hospital	249	3.6	Hospital	5
Turkey—Ankara [98]	January 1998–September 1999	504	23.6	49.4 ^†^	Hospital	610	16.9	Hospital	5
Cyprus [99]	January 1999–December 2005	1109	25.3	40–70	Population	1177	25.0	Population	6
British Women’s Heart and Health Cohort Study UK—Nationwide [100]	April 1999–March 2001	225	25.4	60–79	Population	4061	23.9	Population	5
USA—Arizona, Colorado, Utah, New Mexico [101]	October 1999–May 2004	2303	64.9	<64	Population	2513	59.8	Population	7
Turkey–Istanbul [102]	January 2000–December 2006	1492	18.4	35–70	Hospital	2167	27.8	Clinic	7
Iran—Bandar Abbas [103]	April 2000–March 2002	168	18.5	27–92	Population	504	20.0	Population	6
Malaysia–Kelantan [104]	July 2000–June 2001	147	36.1	26–70	Hospital	147	24.5	Hospital	5
Turkey—Istanbul [105]	September 2002–October 2003	405	23.0	28–72	Hospital	1050	14.7	Hospital	7
Malaysia—Kuala Lumper [106]	July 2004–September 2004	200	34.4	48.7 ^†^	Hospital	183	42.6	Clinic	6
Germany [107]	2004–2005	3593	69.8	n.a.	Population	9098	79.8	Population	5
Iran—Teheran [108]	2004	300	59.0	24–84	Hospital	303	49.2	Hospital	6
Pakistan–Islamabad [109]	January 2005–July 2005	132	18.2	42.4 ^†^	Hospital	145	9.0	Hospital	6

* Participating countries: Australia. Chile. the People’s Republic of China. Colombia. the German Democratic Republic (GDR). Israel. Kenya. Mexico. the Philippines. and Thailand ^†^ mean.

## Data Availability

The datasets used or analyzed during the current study are available from the corresponding author on reasonable request.

## Supplementary Materials

The following are available online at https://www.mdpi.com/article/10.3390/ijerph18094638/s1, Table S1: Quality assessment of included studies based on the Newcastle-Ottawa Scale score.
