# Supplementary material for: Use of Oral Contraceptives as a Potential Risk Factor for Breast Cancer: A Systematic Review and Meta-Analysis of Case-Control Studies Up to 2010
Source: Int J Environ Res Public Health. 2021 Apr 27;18(9):4638. doi: 10.3390/ijerph18094638 (PMC8123798; doi:10.3390/ijerph18094638)
Supplement: Supplementary file 1 [file ijerph-18-04638-s001.zip › ijerph-1161185-supplementary.pdf]

Additional file 1. Quality assessment of included studies based on the Newcastle-Ottawa Scale score

**Study ID**	**Is the case definition adequate**	**Representa tiveness of the cases**	**Selection of the controls**	**Definition of controls**	**Comparability of cases and controls on the basis of the design or analysis**	**Ascertainment of exposure**	**Same method of ascertainment for cases and controls**	**Non-response rate**	**Total stars**
Adebamowo 1999	✱		✱	✱		✱	✱		5
Althuis 2003	✱	✱		✱	✱	✱			5
Beji 2007	✱	✱	✱	✱	✱	✱	✱		7
Bustan 1993	✱	✱		✱	✱			✱	5
Clavel 1991	✱	✱		✱			✱	✱	5
Chie 1998	✱	✱	✱		✱	✱			5
Croghan 2009	✱		✱	✱		✱	✱	✱	6
Dinger 2006	✱	✱		✱		✱	✱		5
Ebrahimi 2002	✱		✱	✱	✱		✱		5
Ellery 1986	✱	✱	✱	✱		✱			5
Ewertz 1992	✱	✱		✱	✱	✱			5
Faheem 2007	✱	✱	✱		✱		✱	✱	6
Gomes 1995	✱	✱		✱	✱	✱			5
Hadjisavvas 2010	✱	✱	✱			✱	✱	✱	6
Hall 2005	✱		✱	✱	✱	✱			5
Harris 1982		✱		✱	✱	✱		✱	5
Harris 1990		✱		✱	✱	✱	✱		5
Hennekens 1984		✱	✱	✱			✱	✱	5
Janerich 1983	✱	✱	✱		✱	✱			5
Jick 1989	✱		✱	✱		✱	✱		5
Kamarudin 2006	✱	✱		✱	✱	✱	✱		6
Kishk 1999	✱	✱		✱	✱	✱			5
Kuru 2002	✱	✱	✱			✱		✱	5

La Vecchia 2006	✱	✱	✱		✱		✱	✱	6
Lawlor 2004		✱		✱	✱	✱	✱		5
Lee 1987	✱	✱	✱		✱	✱			5
Lee 1992	✱	✱	✱		✱	✱			5
Lees 1978	✱	✱		✱	✱			✱	5
Levi 1996	✱	✱	✱		✱	✱		✱	6
Lipworth 1995	✱	✱		✱	✱		✱	✱	6
Lubin 1982	✱	✱		✱			✱	✱	5
Lund 1989	✱	✱	✱		✱	✱			5
Magnusson 1999	✱	✱		✱		✱	✱		5
Mahouri 2007	✱	✱	✱		✱		✱	✱	6
Marchbanks 2002	✱	✱		✱	✱		✱		5
McCredie 1998		✱	✱	✱	✱		✱		5
McPherson 1987	✱		✱	✱	✱			✱	5
Newcomb 1996	✱	✱	✱			✱	✱		5
Norsa'adah 2005	✱	✱		✱	✱	✱			5
Olsson 1989	✱	✱		✱		✱	✱		5
Ozmen 2009	✱	✱	✱	✱	✱		✱	✱	7
Paffenbarger 1980		✱	✱		✱	✱	✱		5
Palmer 1995	✱	✱		✱	✱	✱			5
Paul 1995	✱	✱	✱		✱	✱	✱		6
Pike 1981	✱		✱	✱		✱	✱		5
Price 1999	✱	✱	✱		✱			✱	5
Primic-Žakelj 1995	✱	✱	✱		✱		✱	✱	6
Ravnihar 1988			✱	✱	✱		✱	✱	5
Ravnihar 1979	✱	✱	✱		✱	✱			5
Rohan 1988	✱	✱		✱	✱	✱			5
Rookus 1994	✱	✱	✱		✱	✱		✱	6
Rosenberg 1992	✱	✱		✱	✱		✱		5
Rosenberg 1996	✱	✱	✱	✱	✱		✱	✱	7

Rosenberg 2009	*	*	*	*	*		*	*	7
Rossing 1996	*		*	*	*		*	*	6
Sartwell 1977		*		*	*	*		*	5
Schildkraut 1991	*	*	*		*	*			5
Shantakumar 2007	*	*	*		*		*	*	6
Shapiro 2000	*	*	*		*	*	*	*	7
Stanford 1989	*	*		*		*		*	5
Sweeney 2007	*	*	*		*	*	*	*	7
Talamini 1985		*	*		*		*	*	5
Tavani 1993	*	*	*		*	*			5
Tessaro 2001	*	*	*	*	*			*	6
Traina 1996		*	*		*	*	*		5
UK NCCS 1989	*	*	*	*		*	*		6
Ursin 1998	*	*	*		*		*	*	6
Ursin 1999	*	*	*		*		*	*	6
Vessey 1983		*	*	*		*		*	5
Viladiu 1996	*	*	*		*		*	*	6
Wang 1992	*	*			*		*	*	5
Weinstein 1991	*		*		*		*	*	5
White 1994	*	*	*		*		*	*	6
WHO study 1990	*	*	*	*		*	*	*	7
Wingo 1991	*	*	*		*	*			5
Wynder 1978		*		*	*		*	*	5
Yang 1997	*	*		*		*	*		5
Yavari 2005	*	*	*		*		*	*	6
Yuan 1988	*	*		*		*	*		5
